# Accuracy of emergency physician performed bedside ultrasound in determining gestational age in first trimester pregnancy

**DOI:** 10.1186/2036-7902-4-22

**Published:** 2012-12-06

**Authors:** Turandot Saul, Resa E Lewiss, Marina Del Rios Rivera

**Affiliations:** 1 Department of Emergency Medicine, Emergency Ultrasound Division, St. Luke’s/Roosevelt Hospital Center, 1000 10th Avenue, New York, NY 10019, USA; 2 Department of Emergency Medicine, College of Medicine, University of Illinois at Chicago, 808 South Wood Street, 476C CME, Chicago, IL, 60612, USA

**Keywords:** Gestational age dating, Bedside ultrasound, First trimester pregnancy

## Abstract

**Background:**

Patient reported menstrual history, physician clinical evaluation, and ultrasonography are used to determine gestational age in the pregnant female. Previous studies have shown that pregnancy dating by last menstrual period (LMP) and physical examination findings can be inaccurate. An ultrasound performed in the radiology department is considered the standard for determining an accurate gestational age. The aim of this study is to determine the accuracy of emergency physician performed bedside ultrasound as an estimation of gestational age (EDUGA) as compared to the radiology department standard.

**Methods:**

A prospective convenience sample of ED patients presenting in the first trimester of pregnancy (based upon self-reported LMP) regardless of their presenting complaint were enrolled. EDUGA was compared to gestational age estimated by ultrasound performed in the department of radiology (RGA) as the gold standard. Pearson’s product moment correlation coefficient was used to determine the correlation between EDUGA compared to RGA.

**Results:**

Sixty-eight pregnant patients presumed to be in the 1st trimester of pregnancy based upon self-reported LMP consented to enrollment. When excluding the cases with no fetal pole, the median discrepancy of EDUGA versus RGA was 2 days (interquartile range (IQR) 1 to 3.25). The correlation coefficient of EDUGA with RGA was 0.978. When including the six cases without a fetal pole in the data analysis, the median discrepancy of EDUGA compared with RGA was 3 days (IQR 1 to 4). The correlation coefficient of EDUGA with RGA was 0.945.

**Conclusion:**

Based on our comparison of EDUGA to RGA in patients presenting to the ED in the first trimester of pregnancy, we conclude that emergency physicians are capable of accurately performing this measurement. Emergency physicians should consider using ultrasound to estimate gestational age as it may be useful for the future care of that pregnant patient.

## Background

Pregnant females commonly present to the emergency department (ED) with complaints of pain, bleeding, or other symptoms that necessitate an evaluation to determine the presence of an intra-uterine pregnancy or determine the viability of the pregnancy. The ED is often where the first evaluation of a pregnancy takes place.

Three methods used to estimate gestational age (GA) are menstrual history, clinical examination, and ultrasonography. Previous studies have shown that women cannot provide accurate information about their LMP, can have irregular cycle lengths, and can have irregular ovulation from cycle to cycle, making GA dating calculated by LMP often inaccurate [1,2]. The size of the uterus can be assessed by pelvic examination, but factors such as the presence of fibroids can make this determination difficult. Clinical evaluation by palpation of the gravid uterus increases in accuracy after the first trimester of pregnancy when the uterus rises above the pelvic brim. Ultrasound in the hands of an experience operator is the most accurate means of determining GA [3-6].

First trimester GA is calculated from the gestational sac diameter [7-11] when no fetal pole is yet visible or the fetal crown-rump length (CRL) once it is visualized. The CRL is a sonographic measurement of the length of the fetal pole from the crown to the rump, not including the yolk sac. The following formula can be used: gestational age = 5.2876 + (0.1584 x× CRL) − (0.0007 × CRL^2^); however, most ultrasound machines have a biometric formula software package that can perform this calculation [12]. The correlation between these measurements in the first trimester has been shown to be excellent with an estimated 95% confidence interval of plus or minus 5 days [13]. Several other studies have shown that when CRL is measured between 7 and 10 weeks gestation, the method is accurate within 3 days [14-17]. The first trimester is the optimal time to estimate gestational age when biologic variation in size from fetus to fetus is minimal [18].

Shah et al. reported that emergency physicians can use bedside ultrasound to estimate gestational age in the second and third trimesters of pregnancy as this can be important in trauma and other emergency scenarios [19]. Our study aims to determine the accuracy of emergency physician performed bedside ultrasound as an estimation of gestational age (EDUGA) as compared to the radiology department standard.

## Methods

This was a prospective, convenience sample of pregnant women presenting to the ED for evaluation. Potential participants were identified by the computerized patient tracking system either by stated pregnancy history or by a positive urine pregnancy test. All pregnant women who were thought to be in the first trimester of pregnancy were eligible regardless of their ED presenting complaint. Patients were excluded if they were clinically unstable or required emergent transportation to the operating room, or they were unable or unwilling to give consent. The Institutional Review Board for our hospital approved the study.

The study was performed at a large, urban hospital center with a 3-year emergency medicine residency program. The median age of enrolled patients was 27 years, and 34% identified themselves as African American, 19% as Caucasian, 43% as Hispanic, and 1% as Asian. All residents and attending physicians received training in how to perform a crown-rump length measurement and estimate gestational age through a 30-min didactic lecture and 1-h hands-on ultrasound training session. Prior to the training, all participating attending physicians were credentialed by the department to perform pelvic ultrasound for first trimester of pregnancy indications. Resident physicians had varied previous ultrasound experience based upon their level of training.

**Figure 1:**
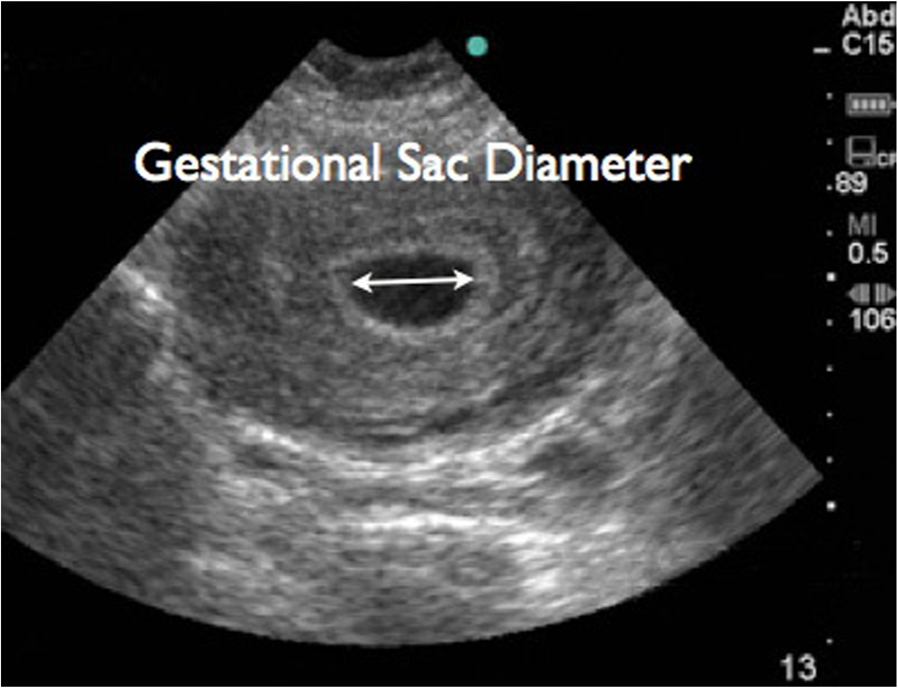
Trans-abdominal ultrasound image showing measurement of gestational sac diameter.

**Figure 2:**
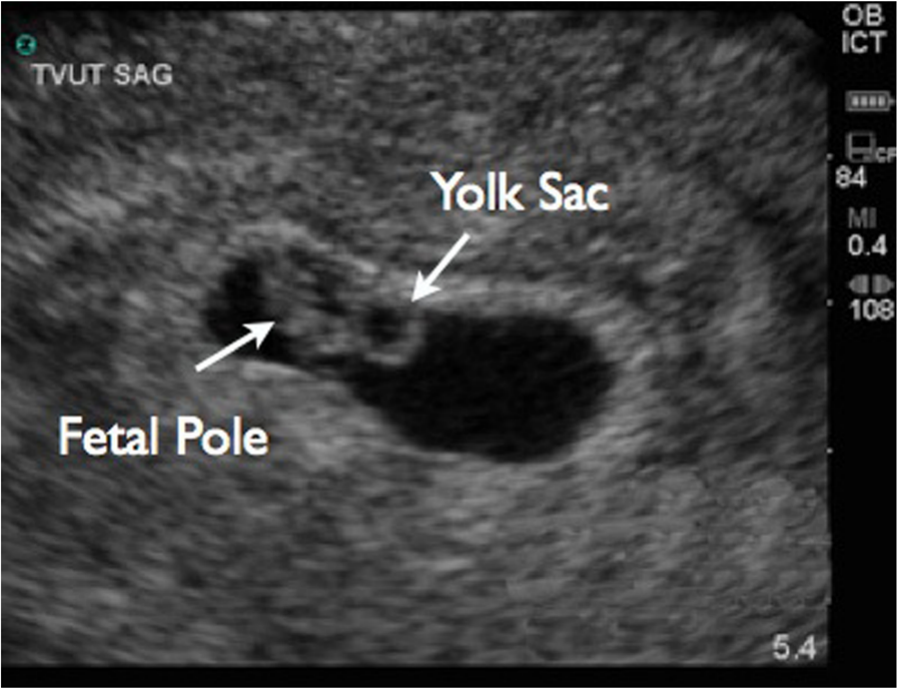
Trans-vaginal ultrasound image showing yolk sac and fetal pole.

**Figure 3:**
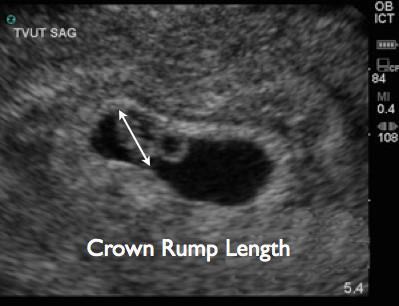
Trans-vaginal ultrasound image demonstrating crown-rump length measurement.

All studies were performed using SonoSite Titan (SonoSite, Inc., Bothell, WA, USA) using either a 2 to 4 MHz tight curvilinear probe for trans-abdominal scanning or a 5 to 8 MHz endocavitary probe for trans-vaginal scanning. Trans-abdominal and/or trans-vaginal ultrasound was performed at the discretion of the attending physician. Measurements were obtained in either a sagittal or transverse plane. In cases where no fetal pole was visualized, caliper measurements were performed of the gestational sac diameter (Figure 1). When a fetal pole was visualized, caliper measurements were performed of the maximal crown-rump length, and the obstetrical software package calculated the GA (Figures 2 and 3). Demographics were collected from the patients as well as their last reported normal LMP.

The level of training (PGY-1, PGY-2, PGY-3, Attending) of the sonographer was recorded. RGA results were collected via real-time or retrospective reporting from the radiologist. Patients presenting to the ED during hours the radiology department ultrasound suite was closed were given instructions to return the following morning for their radiology department ultrasound. The medical record was reviewed for results of these scans performed the following morning. Any patient who did not have their radiology department performed study at our institution was excluded. For all EDUGA scans, thermal images were printed and submitted for review. One investigator reviewed all the EDUGA still images for technical adequacy.

We expected that the EDUGA would have a correlation with the RGA of 0.5. In order to achieve an alpha of 0.01 and beta of 0.05, 62 patients had to be enrolled. We enrolled over a 6-month time period expecting to have some loss to follow up. Statistical analysis was performed using *R* statistical software (version 2.12.1 for Mac; The R Foundation for Statistical Computing, http://www.R-project.org). Our primary outcome measure was the accurate determination of EDUGA using RGA as the gold standard. Pearson’s product moment correlation coefficient was used to determine the correlation between EDUGA and RGA.

## Results

**Figure 4:**
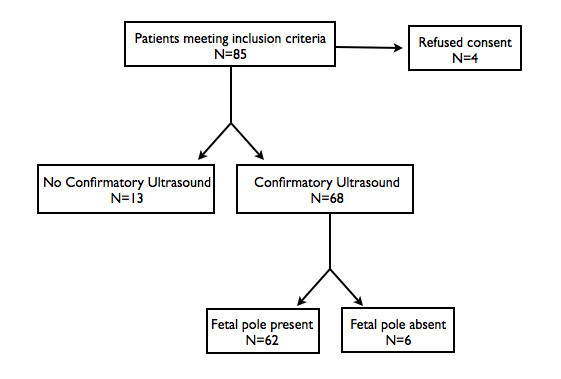
Flow diagram of enrolled patients.

Eighty-five patients met inclusion criteria for enrollment. Four patients did not consent to participate. Eighty-one patients were enrolled in the study. Thirteen patients did not return for a follow-up formal radiology department ultrasound within 24 h of EDUGA after they were seen during hours when the department of radiology was closed. Sixty-eight participants were included in our study (Figure 4). Patient characteristics are listed in Table 1.

**Table 1:** Patient characteristics

**Patient characteristics**	**Number**
Age, years, median (range)	27 (16 to 41)
Gestational age by LMP, weeks, median (IQR)	7 1/7 (6 2/7 to 8 3/7)
Ethnicity, *N *(%)	
African American	23 (34%)
Caucasian	13 (19%)
Hispanic	29 (43%)
Asian	1 (1%)
Not reported	2 (3%)

LMP, last menstrual period; IQR, interquartile range.

Out of the 68, 30 (44%) ultrasounds were performed by attending physicians, 19/68 (28%) by PGY-3 residents, 11/68(16%) by PGY-2 residents, and 8/68 (12%) by PGY-1 residents. Absence of a fetal pole was found in six patients. Absence of a fetal pole does not exclude the possibility of an early intra-uterine pregnancy. It is for this reason that data was analyzed twice. In the first analysis, cases with absence of a fetal pole were excluded (*N* = 62). In the second analysis, all cases were included (*N* = 68).

**Figure 5:**
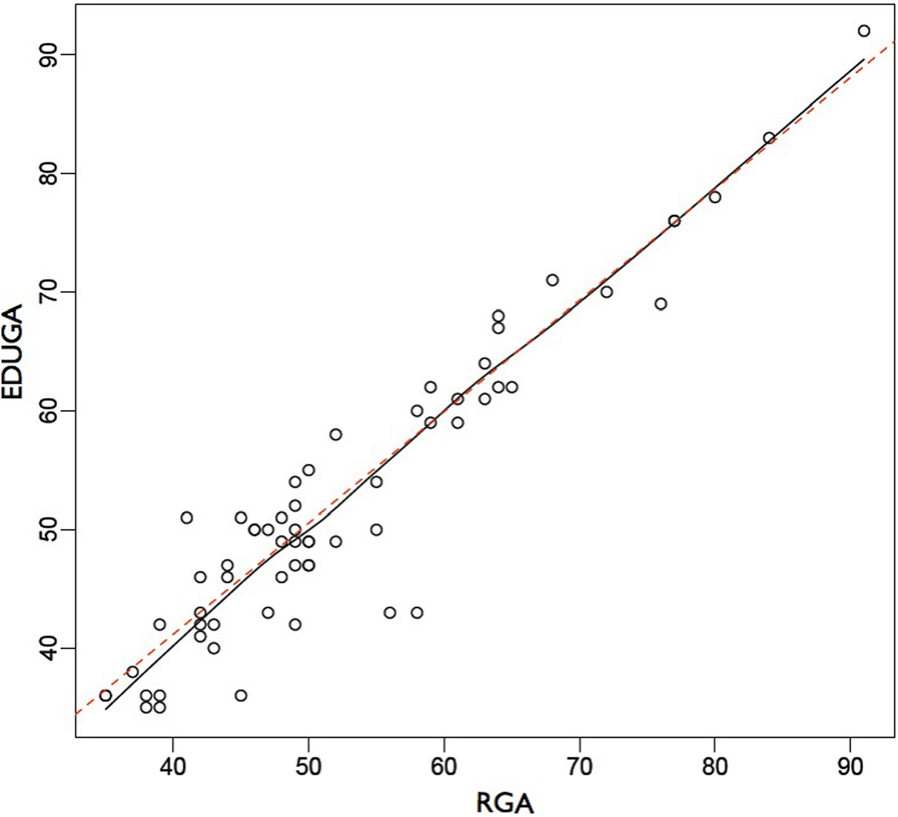
**Scatter plot comparing EDUGA and RGA. **The *x *and *y*-axes measure gestational age in days. Data represented exclude cases in which a fetal pole was not clearly identified. The solid line represents the actual fit; the dashed line is the ideal fit.

When excluding the six cases with no fetal pole, the median discrepancy of EDUGA versus RGA was 2 days (IQR 1 to 3.25) (Table 2). The correlation coefficient of EDUGA with RGA was 0.978. The scatter plot in Figure 5 illustrates the differences between the discrepancies by EDUGA compared to RGA.

**Table 2:** Comparison of EDUGA dating with RGA

	**EDUGA (no fetal pole excluded)**	**EDUGA (all cases)**
Discrepancy with RGA,days, median (IQR)	2 (1 to 3.25)	3 (1 to 4)
Correlation coefficient (95% CI)	0.978 (0.965 to 0986)	0.945 (0.909 to 0.966)
*P *value	*p *< 0.005	*p *< 0.005

IQR, interquartile range; CI, confidence interval.

When including the six cases without a fetal pole in the data analysis, the median discrepancy of EDUGA compared with RGA was 3 days (IQR 1 to 4). The correlation coefficient of EDUGA with RGA was 0.945.

## Discussion

Based on our comparison of EDUGA to RGA in patients presenting to the ED in the first trimester of pregnancy, we conclude that after a brief training, emergency physicians are capable of accurately performing this measurement. This estimation of GA may be more accurate than those made by calculations based on LMP or physical examination findings. The emergency department is, for many, the first time the evaluation of pregnancy takes place, and some patients may not present for routine prenatal care until later in the pregnancy. Since GA dating is most accurate in the first trimester, this may be a useful data point for the future care of that pregnancy. Emergency physicians should consider using ultrasound to estimate gestational age when evaluating these patients.

### Limitations

Physicians that were trained to do these measurements already had a background in pelvic sonography in first trimester pregnancy. At our institution, attending physicians have performed at least 25 previous pelvic sonograms, and residents have exposure early in their residency. Physicians with less prior exposure may require additional training to be able to perform this measurement with a high degree of accuracy. Also, given the convenience sample design of this study, physicians who enrolled patients in the study may have had a greater interest in ultrasound and a higher level of performance than other colleagues who did not enroll patients. In addition, our patient population, age, and ethnicity groups may not be generalizable to other emergency medicine practice populations.

## Conclusion

After a brief training session, emergency physicians are capable of accurately estimating gestational age compared to radiology department ultrasound and should consider performing these measurements in the first trimester of pregnancy.

## Competing interests

The authors declare that they have no competing interests.

## Authors’ contributions

TS was responsible for the study conception and design, data acquisition, and drafting of the manuscript. REL designed the study, acquired the data, and drafted the manuscript. MDR was responsible for supervision of study design, statistical analysis and interpretation, manuscript preparation, and general supervision of the project. All authors read and approved the final manuscript.
